# Diagnostic and Therapeutic Challenge: VEXAS Syndrome with Multisystem Inflammation and Myelodysplasia: A Case Report

**DOI:** 10.31138/mjr.050326.ert

**Published:** 2026-06-16

**Authors:** Bsher Almaalouli, Hannah Smith, Gabriel Kirsch, Gurjit Kaeley

**Affiliations:** 1 Department of Internal Medicine, University of Central Florida College of Medicine, Orlando, FL, United States;; 2 Department of Internal Medicine, HCA Florida North Florida Hospital, Gainesville, FL, United States;; 3 Department of Rheumatology, University of Florida, Jacksonville, FL, United States

**Keywords:** autoinflammatory diseases, myelodysplastic syndromes, anaemia, macrocytic, mutation, somatic, ubiquitin-activating enzymes, haematopoietic stem cell transplantation

## Abstract

VEXAS syndrome (Vacuoles, E1 enzyme, X-linked, Autoinflammatory, Somatic) is an adult-onset autoinflammatory disorder caused by somatic UBA1 mutations linking systemic inflammation with haematologic dysfunction. We report a 59-year-old man presenting with fatigue, fevers, and severe macrocytic anaemia (haemoglobin 8.1 g/dL, MCV >120 fL), mild thrombocytopenia, and markedly elevated inflammatory markers. Autoimmune and malignancy workup was negative. Bone marrow biopsy showed hypercellularity with multilineage dysplasia and cytoplasmic vacuolisation, and genetic testing confirmed a somatic UBA1 mutation. High-dose glucocorticoids led to rapid resolution of inflammatory manifestations and normalisation of inflammatory markers; however, cytopenias progressed despite erythropoiesis-stimulating agents and ruxolitinib, with haemoglobin declining to <7 g/dL, resulting in transfusion dependence. The patient is undergoing evaluation for allogeneic haematopoietic stem cell transplantation (HSCT). This case highlights VEXAS syndrome as an aggressive haematoinflammatory disorder with limited responsiveness to conventional and targeted therapies and emphasises early consideration of advanced strategies, including HSCT, in selected patients.

## INTRODUCTION

VEXAS syndrome is an adult-onset autoinflammatory disorder caused by somatic mutations in the *UBA1* gene, which encodes the E1 ubiquitin-activating enzyme. The acronym stands for Vacuoles, E1 enzyme, X-linked, Autoinflammatory, and Somatic. Because *UBA1* is located on the X chromosome, the syndrome predominantly affects males over age 50, though heterozygous females can also develop symptoms. ^1^

The disease arises from acquired somatic mutations in haematopoietic stem cells involving the UBA1 gene, leading to clonal myeloid expansion. These mutations impair E1 enzyme function and ubiquitination, resulting in defective protein degradation, cytoplasmic vacuole formation, and activation of innate immune pathways.^2^ Clinically, VEXAS presents with both inflammatory and haematologic manifestations. Inflammatory features include recurrent fever, chondritis (particularly auricular and nasal), neutrophilic dermatoses, and pulmonary inflammation. Haematologic abnormalities include macrocytic anaemia, thrombocytopenia, and characteristic cytoplasmic vacuoles in myeloid and erythroid precursors on bone marrow biopsy.^1^

There is currently no established standard of care for VEXAS syndrome. Glucocorticoids remain the primary treatment for controlling inflammatory manifestations; however, their long-term use is limited by significant toxicity. Emerging evidence supports the use of Janus kinase (JAK) inhibitors, IL-6 receptor antagonists, and hypomethylating agents such as azacitidine and decitabine, although responses are variable. Many patients remain refractory to conventional therapies and become dependent on prolonged high-dose glucocorticoids, which significantly increase susceptibility to opportunistic infections, a major contributor to morbidity and mortality in this population.^3^

We report a case of VEXAS syndrome characterised by severe multisystem inflammatory involvement and refractory cytopenias, complicated by glucocorticoid dependence. Despite multiple lines of immunosuppressive and targeted therapy, the patient progressed to transfusion-dependent anaemia and was ultimately referred for allogeneic haematopoietic stem cell transplantation (allo-HSCT).

## CASE DESCRIPTION

We present the case of a 59-year-old man with a history of type 2 diabetes mellitus and hypertension who was admitted in July 2024 with several weeks of progressive fatigue, dizziness, light-headedness, productive cough, intermittent low-grade fevers, and dyspnoea. Initial laboratory evaluation demonstrated anaemia (haemoglobin 8.1 g/dL), marked macrocytosis (MCV >120 fL), mild thrombocytopenia, and markedly elevated inflammatory markers (**Table 1**). During this initial presentation, he reported new-onset painful pustular and nodular skin lesions involving the extremities (**Figure 1**), inflammatory polyarthritis with large joint effusions, and right auricular pain and erythema consistent with chondritis. Computed tomography of the chest, abdomen, and pelvis revealed bilateral pleural effusions, patchy pulmonary infiltrates, mediastinal and abdominal lymphadenopathy, and hepatosplenomegaly, prompting further haematologic evaluation. He was treated with systemic glucocorticoids during hospitalisation, which resulted in rapid improvement of his rash and joint symptoms, though his anaemia persisted. Following hospitalisation, he underwent outpatient lymph node and bone marrow biopsy of which lymph node pathology demonstrated reactive changes without evidence of malignancy. However, bone marrow biopsy revealed a markedly hypercellular marrow with multilineage dysplasia and vacuolisation of erythroid and myeloid precursors without excess blasts.

**Figure 1.**
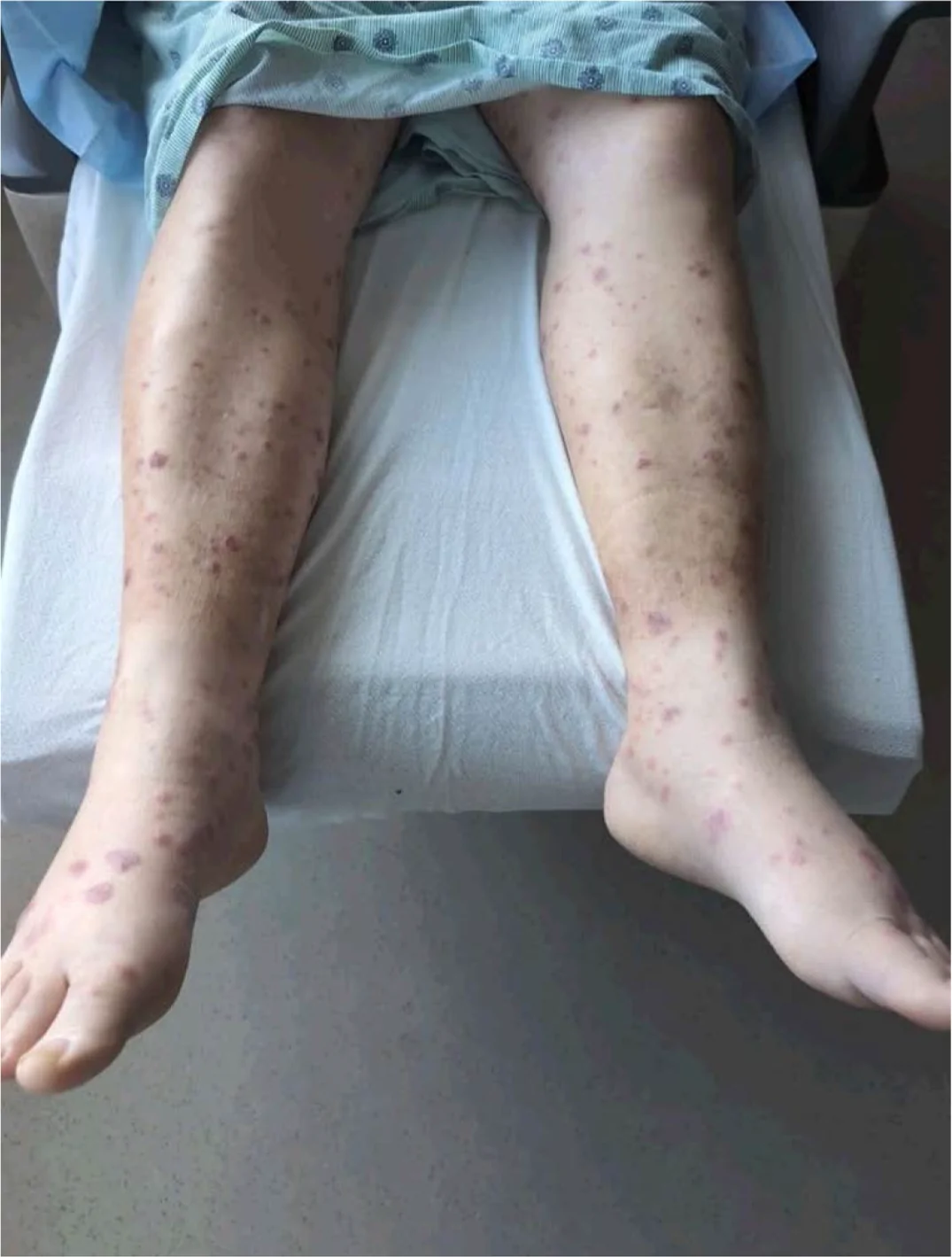
**(left)**. Cutaneous manifestations observed during the patient’s presentation, demonstrating multiple erythematous pustular and nodular lesions on the lower extremities consistent with neutrophilic dermatosis, a recognised cutaneous feature associated with VEXAS syndrome.

**Table 1.** Inflammatory markers.

**Test**	**Value**	**Reference Range**	**Unit**
Haemoglobin	8.1	13.5 – 17.5	g/dL
MCV	124	80 – 100	fL
Platelets	140	150 – 400	×10^9^/L
WBC	8.3	4 – 11	×10^9^/L
Ferritin	1,262	30 – 400	ng/mL
Serum Iron	43	32 – 159	µg/dL
TIBC	154	261 – 390	µg/dL
Transferrin	121	200 – 360	mg/dL
Iron Saturation	28%	20 – 55	–
ANA	Negative	Negative	Negative
Anti-CCP	2	0 – 19	Units
SSA (Ro)	<0.2	0.0 – 0.9	AI
SSB (La)	<0.2	0.0 – 0.9	AI
Uric Acid	3.5	3.5 – 7.2	mg/dL
CRP	168	<10	mg/L

In the setting of adult-onset multisystemic inflammatory manifestations, profound macrocytic anaemia, and bone marrow biopsy findings, he was referred to rheumatology for evaluation of VEXAS syndrome. Targeted genetic testing was performed, which identified a somatic mutation in the *UBA1* gene, specifically, pMet-41Thr, confirming the diagnosis of VEXAS syndrome.

With initiation of high dose glucocorticoids, the patient experienced dramatic response as evidenced by resolution in arthritis, rash, and chondritis. Despite effective control of systemic inflammation, his anaemia progressed. Management was further complicated by the patient’s initial refusal of blood transfusions due to religious beliefs. In the absence of transfusion support, erythropoiesis-stimulating agents were initiated, leading to partial stabilisation of haemoglobin levels. Attempts to taper glucocorticoids were repeatedly unsuccessful, which resulted in prompt disease flares, manifesting as facial and eyelid swelling, arthralgias, and constitutional symptoms. Recurrent flares led to multiple hospitalisations and repeated courses of intravenous steroids. Given the high cumulative steroid exposure and associated complications, including Cushingoid features, brittle glycaemic control, and infection risk, a steroid-sparing strategy was pursued in collaboration with haematology.

The patient was started on ruxolitinib, which initially allowed modest steroid dose reduction. However, it was complicated by worsening pancytopenia and a severe respiratory infection requiring hospitalisation, that led to temporary discontinuation of the JAK inhibitor. During periods of steroid interruption or reduction for infection management, the patient again experienced inflammatory flares. Over time, he became increasingly dependent on prednisone 40 mg daily to maintain partial inflammatory control.

As the disease course progressed, the patient developed persistent transfusion-dependent anaemia secondary to myelodysplasia. He ultimately accepted intermittent transfusions in the setting of symptomatic, life-threatening anaemia, along with continued use of erythropoiesis-stimulating agents.

Despite trials of immunosuppression and targeted therapy, the patient remained steroid-dependent with refractory haematologic disease. He was referred for evaluation of allo-HSCT. Given the failure of conventional and targeted immunosuppressive strategies, and in light of emerging evidence supporting HSCT as a potentially curative option for VEXAS syndrome, he was deemed an appropriate candidate. At the time of transplant evaluation, he was receiving prednisone, low-dose ruxolitinib, erythropoietic support, and had recently initiated imetelstat for MDS-related cytopenias. His inflammatory disease was clinically controlled on high-dose steroids. At the time of writing, the patient is undergoing pre-transplant evaluation for allo-HSCT, with careful tapering of glucocorticoids under close rheumatologic and haematologic follow-up.

## DISCUSSION

This case adds to the growing recognition of VEXAS syndrome as a heterogeneous haematoinflammatory disorder characterised by multisystem inflammation, macrocytic anaemia, and bone marrow dysplasia. Our patient highlights the diagnostic complexity and therapeutic challenges of VEXAS, including profound glucocorticoid dependence and progressive haematologic involvement. Key clinical red flags include the coexistence of inflammatory manifestations such as recurrent fever, chondritis (especially auricular and nasal), neutrophilic dermatoses, pulmonary infiltrates, venous thrombosis and inflammatory arthritis with haematologic abnormalities.^1^ Large cohort analyses demonstrate that VEXAS predominantly affects older men and is characterised by multisystem involvement. The most common manifestations include haematologic abnormalities (≈87%), particularly macrocytic anaemia and cytopenias, followed by dermatologic involvement (≈82%) such as neutrophilic dermatoses, pulmonary disease (≈75%) including ground-glass opacities and pleural effusions, and musculoskeletal manifestations (≈44%) such as inflammatory arthritis. Constitutional symptoms, especially fever (≈68%), are also highly prevalent. These findings are consistent with the clinical presentation observed in our patient, who exhibited both systemic inflammation and progressive haematologic dysfunction.^4^

Unlike classic rheumatic diseases such as polymyalgia rheumatica or seronegative rheumatoid arthritis, VEXAS follows an atypical course marked by resistance to conventional immunosuppressive agents and TNF inhibitors, evolving bone marrow failure, and sustained steroid dependence. Chondritis in VEXAS, while clinically resembling relapsing polychondritis, is generally non-destructive and typically spares the airways and costochondral junctions—features that help distinguish it from classic relapsing polychondritis.^5^

Pulmonary involvement is common and characterised by interstitial infiltrates on chest computed tomography, most frequently ground-glass opacities (87%), consolidations (49%), septal lines (51%), and pleural effusions (53%). The presence of macrocytic anaemia or macrocytosis, thrombocytopenia, and elevated inflammatory markers should particularly raise suspicion of VEXAS.

Bone marrow findings provide critical diagnostic clues. Characteristic cytoplasmic vacuoles in myeloid and erythroid precursors on bone marrow biopsy are highly suggestive, often accompanied by hypercellularity and multilineage dysplasia. Definitive diagnosis requires detection of somatic UBA1 mutations through genetic testing. Misdiagnosis is common, with VEXAS overlapping with other autoimmune and haematologic diseases such as Still’s disease, relapsing polychondritis, and myelodysplastic syndromes.^1^ Renal involvement in VEXAS syndrome, once thought to be rare, is increasingly recognised, occurring in approximately 3–20% of patients and up to 39% in long-term follow-up. Clinical presentations include acute kidney injury, chronic kidney disease, protein-uria, and haematuria, with interstitial nephritis being the most commonly reported pathology, along with various glomerular diseases. The underlying mechanism is likely multifactorial, involving both direct infiltration by UBA1-mutant myeloid cells and immune-mediated injury. Renal manifestations often parallel systemic inflammation and respond to glucocorticoids, though relapses are common. In our case, the absence of renal involvement highlights the variable and heterogeneous nature of VEXAS syndrome.^6^ The diagnostic journey highlights several important challenges in recognising VEXAS syndrome. Although adult-onset multisystem inflammation accompanied by severe macrocytic anaemia should raise suspicion for VEXAS, diagnosis is frequently delayed by the presence of low-titre or nonspecific positive autoimmune serologies, which can be misleading. As a result, patients are often initially diagnosed with more common rheumatic or inflammatory conditions, including relapsing polychondritis, systemic vasculitis, or adult-onset Still’s disease. Bone marrow findings served as a critical diagnostic clue in this case, as they do in most VEXAS patients. The prognosis of VEXAS syndrome remains poor, with mortality rates of 15–40% at 5 years in published cohorts. The p.Met41Val variant is associated with decreased survival compared to p.Met41Leu or p.Met41Thr mutations, and transfusion dependence independently predicts poor outcomes. Infections represent the leading cause of death, accounting for the majority of fatal events, followed by progression of haematologic disease.^7^ Corticosteroids remain first-line therapy for inflammatory manifestations, with patients typically responding to prednisone ≥20 mg daily. However, profound steroid dependence is characteristic, with disease flares occurring upon tapering below 20 mg daily, and cumulative toxicity contributing substantially to morbidity. Glucocorticoid dependence represents the most significant therapeutic challenge in VEXAS syndrome. As demonstrated in this case, while high-dose corticosteroids consistently achieve dramatic inflammatory control, attempts to taper below 20–40 mg daily prednisone equivalents typically trigger disease flares. The partial efficacy of ruxolitinib in this case aligns with recent evidence regarding JAK inhibitors in VEXAS, though treatment can be complicated by worsening cytopenias and infectious complications.^5^ IL-6 inhibitors such as tocilizumab and sarilumab can effectively control inflammatory manifestations and facilitate glucocorticoid dose reduction in many patients, though most experience progressive bone marrow failure despite inflammatory control. In larger cohort analyses, glucocorticoids were used in the majority of patients, followed by methotrexate and IL-6 inhibitors, highlighting their role as common therapeutic strategies in VEXAS syndrome.^4^ IL-1 inhibitors, including anakinra and canakinumab, have shown efficacy in selected cases, with case series demonstrating steroid-sparing effects and clinical stabilisation in some patients.^2^ In this patient, the progression to transfusion-dependent anaemia reflected worsening haematologic disease despite adequate control of inflammatory manifestations. This phenomenon reflects the underlying clonal haematopoiesis and bone marrow failure inherent to VEXAS pathophysiology. The limited response to erythropoiesis-stimulating agents is typical, as these therapies do not address the fundamental defect in UBA1-mutated haematopoietic stem cells. Allo-HSCT represents the only curative therapeutic option for VEXAS syndrome. Recent analyses demonstrate that allo-HSCT can achieve complete eradication of the UBA1 mutation, though the procedure carries significant risks including non-relapse mortality, graft-versus-host disease (GVHD), and infectious complications. Effective control of inflammation prior to transplantation appears essential for optimal outcomes. In this case, the decision to pursue allo-HSCT was based on careful assessment of transplant candidacy. Key factors supporting transplant referral include refractory disease despite multiple therapeutic lines, cumulative steroid toxicity, transfusion dependence, and relatively young age with acceptable performance status. The American College of Rheumatology guidance suggests that allo-HSCT should be considered for younger, fit patients with poor prognostic factors or recalcitrant symptoms.^1^

As summarised in **Table 2**, published cases of VEXAS undergoing HSCT demonstrate that patients typically have refractory disease after multiple therapies, and while HSCT offers the potential for durable remission, it is associated with significant risks. Our patient follows a similar trajectory, with progressive cytopenias and steroid dependence despite multiple therapies, supporting early consideration of transplant evaluation.

**Table 2.** Published cases of VEXAS undergoing HSCT.

**Study**	**Patient Demographics**	**Clinical Presentation**	**Prior Therapies**	**HSCT Details**	**Outcomes**
Diarra et al. (2022), 2-center retrospective series^8^	Older males with UBA1 mutations	Refractory autoinflammatory disease; cytopenias, MDS features	Multiple failed immunosuppressive therapies	Allogeneic HSCT (variable conditioning)	5/6 achieved remission; 1 death
Ali Gurnari (2024), systematic review of published cases^9^	97% male; median age 59 years	Systemic inflammation, cytopenias, MDS; most common UBA1 mutation: Met41Thr (34.4%); median VAF 56.5%	Median 4.5 prior therapies (steroids, JAKi, immunosuppressants)	PB-HSCT (97%); mostly matched unrelated donors; reduced-intensity conditioning common	81.8% alive; UBA1 eradication in tested patients; GVHD 56%; infections 37% implicated in 4)
Mohty et al. (2024), systematic review and meta-analysis^10^	Adult-onset, predominantly male (5th decade or later)	Refractory inflammatory disease with marrow dysfunction	Multiple prior therapies	Allogeneic HSCT (heterogeneous protocols)	OS 86%; EFS 56%; NRM 14%; GVHD common; no relapse reported

## CONCLUSION

This case illustrates the classic presentation of VEXAS syndrome, characterised by adult-onset multisystem inflammation, severe macrocytic anaemia, and marked steroid dependence. Recognition of this entity is critical in older patients with unexplained inflammatory manifestations, cytopenias, negative autoimmune serologies, and characteristic bone marrow findings. Although glucocorticoids provide rapid and effective control of inflammatory symptoms, long-term disease management is limited by profound steroid dependence, treatment-related toxicity, and progressive haematologic failure.

Targeted therapies may offer partial benefit but are frequently constrained by cytopenias and infectious complications. The progression to transfusion-dependent anaemia reflects the underlying clonal bone marrow disorder and the limited benefit of immunosuppressive therapy alone. In this context, allogeneic haematopoietic stem cell transplantation remains the only potentially curative option and should be considered early in appropriate candidates. Multidisciplinary collaboration between rheumatology and haematology is essential to optimise outcomes and minimise diagnostic delay in this complex disorder.
